# Size is an essential parameter in governing the UVB-protective efficacy of silver nanoparticles in human keratinocytes

**DOI:** 10.1186/s12885-015-1644-8

**Published:** 2015-09-15

**Authors:** Rohan Palanki, Sumit Arora, Nikhil Tyagi, Lilia Rusu, Ajay P. Singh, Srinivas Palanki, James E. Carter, Seema Singh

**Affiliations:** 1 Department of Oncologic Sciences, Mitchell Cancer Institute, University of South Alabama, 1660 Springhill Avenue, Mobile, AL 36604 USA; 2 Department of Chemical and Biomolecular Engineering, University of South Alabama, Mobile, AL 36688 USA; 3 Department of Biochemistry and Molecular Biology, College of Medicine, University of South Alabama, Mobile, AL 36688 USA; 4 Department of Pathology, College of Medicine, University of South Alabama, Mobile, AL 36688 USA

## Abstract

**Background:**

Ultraviolet (UV) radiation from sun, particularly its UVB component (290–320 nm), is considered the major etiological cause of skin cancer that impacts over 2 million lives in the United States alone. Recently, we reported that polydisperse colloidal suspension of silver nanoparticles (AgNPs) protected the human keratinocytes (HaCaT) against UVB-induced damage, thus indicating their potential for prevention of skin carcinogenesis. Here we sought out to investigate if size controlled the chemopreventive efficacy of AgNPs against UVB-induced DNA damage and apoptosis.

**Methods:**

Percent cell viability was examined by WST-1 assay after treating the cells with various doses (1–10 μg/mL) of AgNPs of different sizes (10, 20, 40, 60 and 100 nm) for 12 and 24 h. For protection studies, cells were treated with AgNPs of different sizes at a uniform concentration of 1 μg/mL. After 3 h, cells were irradiated with UVB (40 mJ/cm^2^) and dot-blot analysis was performed to detect cyclobutane pyrimidine dimers (CPDs) as an indication of DNA damage. Apoptosis was analyzed by flow cytometry after staining the cells with 7-Amino-Actinomycin (7-AAD) and PE Annexin V. Immunoblot analysis was accomplished by processing the cells for protein extraction and Western blotting using specific antibodies against various proteins.

**Results:**

The data show that the pretreatment of HaCaT cells with AgNPs in the size range of 10–40 nm were effective in protecting the skin cells from UVB radiation-induced DNA damage as validated by reduced amounts of CPDs, whereas no protection was observed with AgNPs of larger sizes (60 and 100 nm). Similarly, only smaller size AgNPs (10–40 nm) were effective in protecting the skin cells from UV radiation-induced apoptosis. At the molecular level, UVB –irradiation of HaCaT cells led to marked increase in expression of pro-apoptotic protein (Bax) and decrease in anti-apoptotic proteins (Bcl-2 and Bcl-xL), while it remained largely unaffected in skin cells pretreated with smaller size AgNPs (10–40 nm).

**Conclusions:**

Altogether, these findings suggest that size is a critical determinant of the UVB-protective efficacy of AgNPs in human keratinocytes.

## Background

Skin cancer is the most commonly diagnosed malignancy in the United States of America [1]. Each year, over 2 million new cases of skin cancer are diagnosed, which is greater than the combined incidence of cancers of the breast, prostate, lung and colon [1, 2]. Ultraviolet B (UVB) radiation has been well established as one of the strongest etiologic risk factors responsible for the occurrence of skin cancers [3, 4]. Direct exposure of skin to UVB radiation causes DNA damage in skin cells, due to formation of cyclobutane pyrimidine dimers (CPDs), cytosine photohydrates, and purine photoproduct [5, 6]. Furthermore, UVB radiation leads to reactive oxygen species (ROS) generation in exposed cells that results in oxidative DNA damage, including single and double-strand breaks and generation of 8-hydroxyl-2-deoxyguanine (8-OHdG) [7, 8]. If a cell fails to repair this DNA damage, it could accumulate carcinogenic mutations, causing a malignant transformation.

The traditional approach to protect against the harmful effects of UV-radiations is to apply sunscreen lotion as a direct barrier on the skin. Even though sunscreens have been used since 1928, they have failed in limiting the UV-induced skin cancer occurrence [9]. Sunscreens are formulated to contain UV filters or reflectors, such as zinc oxide nanoparticles and titanium dioxide nanoparticles [9, 10]. However, recent studies have shown that zinc oxide nanoparticles and titanium dioxide nanoparticles can have inflammatory/toxic effects on normal skin cells [11, 12].

Silver nanoparticles (AgNPs) are emerging as one of the fastest growing nanotechnology-based product categories [13–15]. This has led to increasing number of medical applications of silver nanoparticles. It is estimated that more than 30 % of nanotechnology-based products contain AgNPs [16]. AgNPs containing products include surgical instruments, wound dressings, contraceptive devices, water purification devices etc., thus suggesting their widespread applications in nanomedicine [17–22].

Recently, it has been shown that polydisperse colloidal suspension of silver nanoparticles protect the human keratinocytes against UVB-induced damage and have potential for prevention of skin carcinogenesis [23]. Although bulk materials have constant physicochemical properties regardless of its size, however at the nano-scale, size is one of the important criteria that governs the physicochemical properties and ultimately depict their biological behavior. Therefore, in the present study we have explored the effects of different sizes of AgNPs against UVB radiation-induced DNA damage. Our studies reveal that AgNPs are non-toxic to human keratinocytes in the size range of 1–100 nm (Concentrations 1–10 μg/mL). Our data demonstrate that AgNPs in the size range 10–40 nm are able to protect human keratinocytes (HaCaT) from UVB-induced DNA damage and also significantly reduce the extant of apoptosis caused by UVB radiation. Furthermore, our data indicates that the pro-apoptotic proteins are down-regulated and the anti-apoptotic proteins are up-regulated in the presence of silver nanoparticles of smaller size (10 nm), whereas no such changes have been observed at 100 nm AgNPs. These are promising observations and provide valuable information on the size-dependent protective effects for potential human applications against UVB-induced skin carcinogenesis.

## Methods

### Reagents

Dulbecco's modified Eagle's medium (DMEM) was obtained from Thermo Fisher Scientific (Logan, UT). Fetal-bovine serum (FBS) was purchased from Atlanta Biologicals (Lawrenceville, GA). Penicillin, streptomycin and trypsin-EDTA were ordered from Invitrogen (Carlsbad, CA). Silver nanoparticles with average diameter of 10, 20, 40, 60 and 100 nm were obtained from Sigma-Aldrich (St. Louis, MO). Trypan blue was received from Thermo Fisher Scientific (Logan, UT) and cell counting chamber slides were acquired from Life Technologies (Grand Island, NY), respectively. Tris buffered saline (TBS) was purchased from Boston Bioproducts (Ashland, MA) and Tween-20 was obtained from Fisher BioReagents (Pittsburgh, PA). Cell proliferation reagent (WST-1) was received from Roche Diagnostics (Mannheim, Germany) and PE Annexin V apoptosis detection kit was ordered from BD Bioscience (San Diego, CA). DNAzol was obtained from Molecular Research Center (Cincinnati, OH), and antibody against Cyclobutane pyrimidine dimer (mouse monoclonal) was purchased from KamiYa Biomedical Company (Seattle, WA). Anti-Bcl-2 (rabbit monoclonal), -Bcl-xL, -Bax (rabbit polyclonal) antibodies were purchased from Cell Signaling Technology (Beverly, MA). The mouse monoclonal β-actin was procured from Sigma-Aldrich. All respective anti-rabbit and anti-mouse horseradish peroxidase-conjugated secondary antibodies were obtained from Santa Cruz Biotechnology (Santa Cruz, CA).

### Cell culture

HaCaT, an immortal non-cancerous human keratinocyte cell line (German Cancer Research Center, Heidelberg, Germany) was employed in this study. HaCaT cells were cultured in DMEM medium containing 2 mM L-glutamine and supplemented with 10 % fetal bovine serum (FBS), penicillin (100 units/mL) and streptomycin (100 μg/mL) at 37 °C in 5 % CO_2_. Mycoplasma testing of cells was performed intermittently using Mycosensor PCR assay kit (Stratagene, La Jolla, CA).

### Cell viability assay

Percent growth cell viability was examined through the WST-1 (4-[3-(4-iodophenyl)-2-(4-nitrophenyl)-2H-5-tetrazolio]-1, 3-benzene di-sulfonate) assay kit, as described earlier [24]. HaCaT cells were seeded in a 96-well plate (1 × 10^4^ cells/well) and incubated overnight. Cells were treated with various concentrations (1–10 μg/mL) of different size silver nanoparticles (10, 20, 40 60 and 100 nm). Following further incubation for 12 and 24 h, supernatants were aspirated out, cell monolayers were washed with PBS, and WST reagent (100 μL) was added in each well. Cells were incubated for 1 h and the absorbance was measured at a wavelength of 450 nm (reference wavelength 630 nm) using a Bio-Rad Benchmark microplate reader. Percent viability was calculated using the formula (absorbance of treated cells)/ (absorbance of control cells) × 100.

### Dot-blot analysis

HaCaT cells were seeded (1 × 10^6^ cells/plate) in glass plates and incubated overnight. Cells were treated with different sizes (10, 20, 40, 60 and 100 nm) of silver nanoparticles at a concentration of 1 μg/mL. After 3 h, cells were irradiated with UVB (40 mJ/cm^2^) using a Daavlin Research Irradiator (Bryan, OH) equipped with four UVB lamps and an electronic controller to regulate UVB dosage. A majority of the wavelengths of UVB radiation were in the 280–320 nm range. Genomic DNA (treated or untreated) was isolated using DNAzol, as per manufacturer’s instructions, and transferred (500 ng) to a positively charged nitrocellulose membrane. Immobilized DNA was fixed by baking the membrane for 30 min at 80 °C. To avoid non-specific binding, a blocking buffer (5 % non-fat dry milk, 1 % Tween-20 in 20 mM TBS, pH 7.6) was utilized. Subsequently, the membrane was incubated, with an anti-CPDs antibody, for 2 h at room temperature, followed by washing with TBST (Tris buffered saline with 0.05 % Tween 20) and incubation with HRP-conjugated secondary antibody. The membrane was processed with an ECL plus detection kit (Thermo Scientific, Logan, UT) and the signal was detected using an LAS-3000 image analyzer (Fuji Photo Film Co., Tokyo, Japan).

### Apoptosis assay

HaCaT cells were seeded (1 × 10^6^ cells/plate) and treated with UVB radiation as described above. The cells were harvested 24 h after UVB exposure and stained with 7-Amino-Actinomycin (7-AAD) and PE Annexin V, using the PE Annexin V Apoptosis Detection Kit I as described earlier [25, 26]. The stained cells were analyzed by flow cytometry on a BD-FACS Canto^TM^ II (Becton-Dickinson, San Jose, CA). Percentage of apoptotic cell population was calculated using Mod Fit LT software (Verity Software House, Topsham, ME) and compared with appropriate controls.

### Immunoblot analysis

Cells were processed for protein extraction and Western blotting as described earlier [27, 28] using specific antibodies against various proteins. Primary antibodies were used at 1:1000 dilutions, whereas all secondary antibodies were used at 1:2000 dilution. β-actin was used as loading control at a dilution of 1:20000. Blots were processed with Enhanced chemiluminescence (ECL) plus Western blotting detection kit and the signal detected using an LAS-3000 image analyzer (Fuji Photo Film Co).

### Statistical analysis

Each experiment was performed at least three times, independently. All the values were expressed as mean ± standard deviation. Wherever appropriate, the data were also subjected to unpaired two tailed Student's *t*-test. A value of *p* < 0.05 was considered as significant.

## Results

### Silver nanoparticles are largely non-toxic to human keratinocytes (HaCaT)

**Fig. 1:**
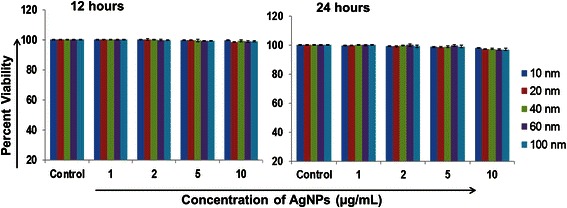
Silver nanoparticles (10–100 nm) are not cytotoxic to human keratinocytes. HaCaT cells grown in 96 well plates (1×10^4^ cells/well) were treated with AgNPs (1–10 μg/mL) of different size (10, 20, 40 and 100 nm) for 12 and 24 h. After treatment, the percent viability of cells was measured by WST-1 assay as per manufacturer’s instructions. The absorbance value of control cells was taken as 100 % viable and percent viability was calculated using the formula (absorbance of treated cells)/ (absorbance of control cells) × 100. Data are expressed as mean ± SD; (*n* = 3)

To examine the toxicity of AgNPs, HaCaT cells were treated with AgNPs (size-10, 20, 40, 60 and 100 nm) at various concentrations (1, 2, 5 and 10 μg/ml) for 12 and 24 h and the percent viabilities were measured by WST-1 assay. Cell viability of HaCaT cells was observed to be over 96 % as compared to untreated cells at all the sizes and maximum concentration (10 μg/mL) upto 24 h (Fig. 1). Together, these results demonstrate that AgNPs in the size range of 10–100 nm and the concentration range of 1–10 μg/ml are largely non-toxic to human keratinocytes.

### Silver nanoparticles in the size range of 10–40 nm protect human keratinocytes from UVB-induced DNA damage

**Fig. 2:**
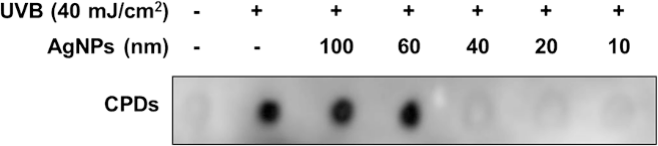
Silver nanoparticles of smaller size (10–40 nm) are effective in reducing and or repairing the formation of CPDs in UVB-exposed human Keratinocytes. HaCaT cells (1×10^6^/plate) seeded in UV transparent glass plates were treated with AgNPs (1 μg/mL) of different size (10–100 nm) for 3 h prior to UVB-exposure (40 mJ/cm^2^). After 24 h, genomic DNA was isolated and subjected to dot-blot analysis using an antibody specific to CPDs

UVB radiations upon direct exposure to skin result in DNA damage of skin cells due to the formation of cyclobutane pyrimidine dimers (CPDs), a major class of UVB-induced harmful DNA lesions [5]. In a recent study AgNPs have been shown to protect human keratinocytes against UVB-induced DNA damage [23]. To determine the effect of size of AgNPs on the protection of HaCaT cells from UVB-induced DNA damage, we examined the formation of CPDs. The HaCaT cells were treated with AgNPs (1 μg/mL) of different size before irradiation to UVB (40 mJ/cm^2^) and CPDs formation was investigated by dot-blot assay. UVB-irradiated cells without AgNPs pretreatment served as positive control for CPDs formation. Our data indicated that HaCaT cells treated with 10, 20 and 40 nm AgNPs prior to UVB-irradiation had no CPD formation suggesting the protection against UVB-induced DNA damage (Fig. 2). However, CPDs formation was observed in the HaCaT cells treated with 60 and 100 nm AgNPs prior to UVB-irradiation (Fig. 2). Altogether, these findings suggest that AgNPs protect HaCaT cells from UVB-induced DNA damage in the size range of 10–40 nm and no protection is observed at size ≥ 60 nm.

### UVB-induced apoptosis is inhibited by silver nanoparticles in the size range of 10–40 nm

**Fig. 3:**
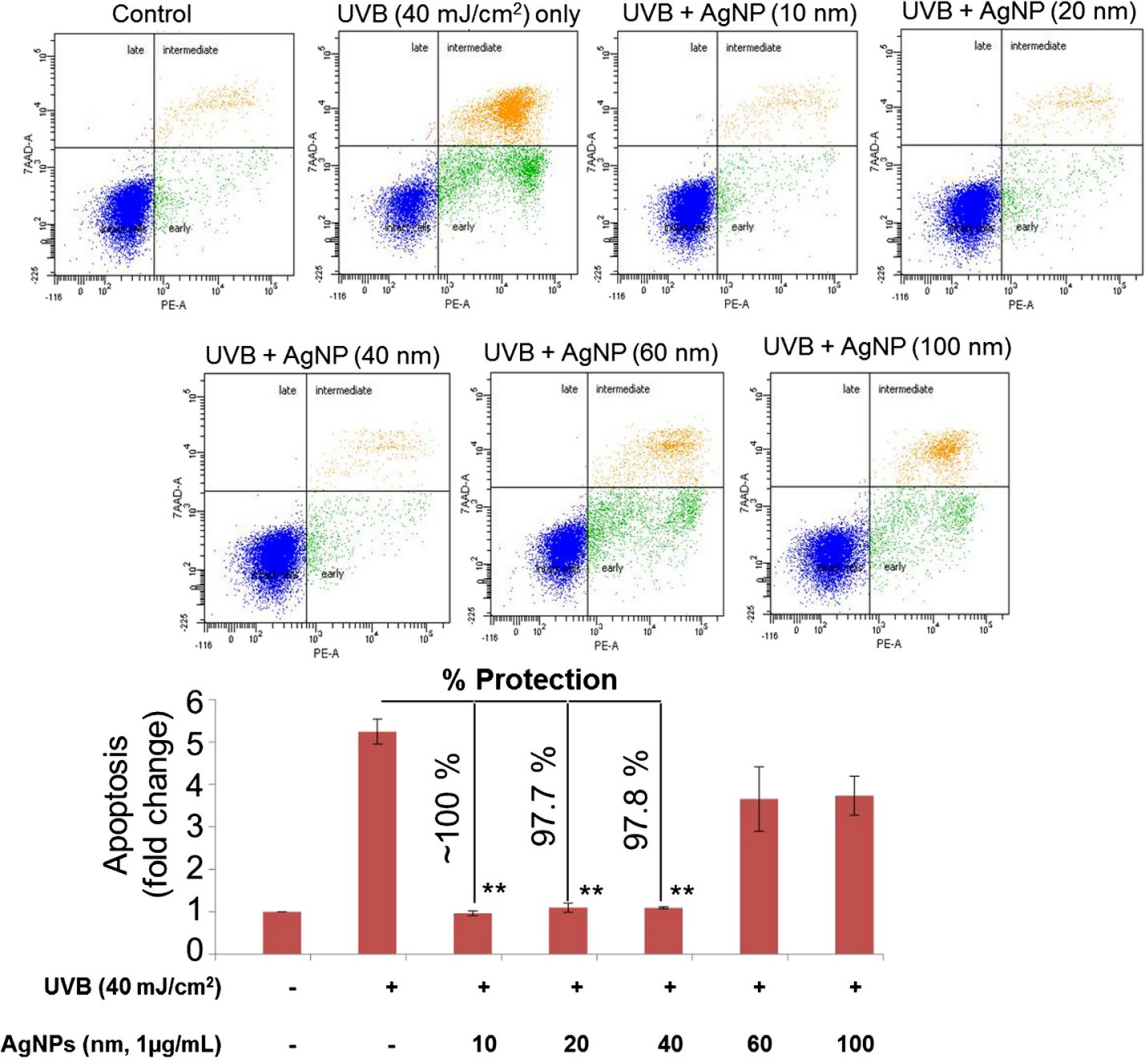
Silver nanoparticles in the size range of 10–40 nm protect human keratinocytes from UVB-induced apoptosis. HaCaT cells (1×10^6^ /plate) seeded in UV transparent glass plates were pretreated with AgNPs for 3 h before UVB-exposure (40 mJ/cm^2^). Untreated and UVB unexposed cells were used as controls. After 24 h post UVB-exposure, cells were harvested, stained with PE Annexin V and 7AAD and analyzed by flow cytometry. The percentage of early apoptotic cells in each treatment group was calculated. Bars represent mean ± standard deviation, *n* = 3

**Fig. 4:**
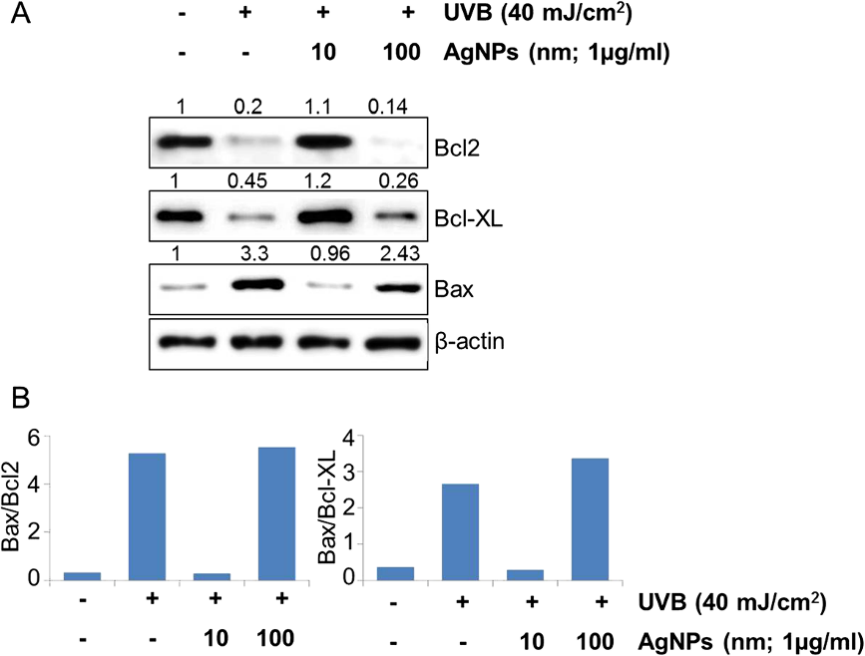
Treatment with smaller size AgNPs modulates the expression of proteins related to survival of human keratinocytes. **a** HaCaT cells (1×10^6^/plate) were seeded in UV transparent glass plates for 70 % confluence. Thereafter, cells were pretreated with AgNPs for 3 h prior UVB-exposure (40 mJ/cm^2^). After 24 h post UVB-exposure, total protein was isolated and subjected to immunoblot analysis for cell survival related proteins namely Bcl-xl, Bcl-2 and Bax followed by densitometry of immunoreactive bands. Normalized densitometric values are indicated at the top of the bands. β-actin was used as a loading control. **b** Bar diagram represents the Bax/Bcl-2 and Bax/Bcl-xL ratio in different treatment groups

To further study the effect of size on UVB-induced cell death, apoptosis studies were conducted with HaCaT cells after treatment with AgNPs. The HaCaT cells were exposed to UVB radiations (40 mJ/cm^2^) after pre-treatment with AgNPs at a concentration of 1 μg/mL. Our data demonstrated a considerable increase in apoptotic index (PE Annexin V positive/7AAD negative cells) after UVB-exposure (Fig. 3). After treatment with AgNPs of different size, we observed that the nanoparticles in size range 10–40 nm were fully efficient in protecting the HaCaT cells from UVB-induced apoptosis. The percent protection by 10 nm AgNPs was almost 100 % while AgNPs of size 20 and 40 nm were able to protect cells from UVB-induced apoptosis by ~97.7 and ~ 97.8 5, respectively (Fig. 3). On contrary, AgNPs of size 60 and 100 nm were not effective as smaller size AgNPs (Fig. 3). To investigate the molecular basis of protection against UVB-induced apoptosis, we examined the effect on expression of key proteins involved in cell survival. We observed that UVB irradiation causes a reduction in the levels of the anti-apoptotic protein Bcl-2 and Bcl-xL, whereas an associated increase in the level of pro-apoptotic protein Bax was observed (Fig. 4a) leading to an increase in the ratio of Bax/Bcl-2 and Bax/Bcl-xL (Fig. 4b). However, relatively similar expression of these proteins was observed in AgNPs (10 nm) pre-treated HaCaT cells prior to UVB-irradiation (Fig. 4a) in comparison to UVB-untreated cells. Interestingly, expression of these proteins in HaCaT Cells pre-treated with 100 nm AgNPs, prior to UVB-exposure caused similar changes in the expression of anti-apoptotic and pro-apoptotic proteins as that of UVB-exposed cells (Fig. 4a). These findings demonstrate that smaller size AgNPs (10 nm) alters the expression of proteins involved in the regulation apoptosis to confer its protective effects, whereas no such modulations have been observed with higher size (100 nm) of AgNPs.

## Discussion

The incidence of skin cancer has been increasing at an alarming rate over the past several decades, and it is anticipated that over 1 million new cases of skin cancer arise each year in the United States [2, 29]. Ultraviolet radiation has been identified as the major environmental carcinogen in skin cancer. Recently, AgNPs have been shown to be protective against UVB radiation-induced DNA damage and apoptosis [23]. To fully explore the potential of AgNPs for chemopreventive applications against UVB-irradiation, the current study was undertaken to investigate the protective effects of a well-characterized panel of AgNPs with a specific focus on size.

UVB radiation directly damages the DNA of skin cells, causing DNA lesions. One of the most lethal DNA lesions induced by UVB radiation is cyclobutane pyrimidine dimers (CPDs). We observed that treatment of HaCaT cells with small size AgNPs (size range 10–40 nm) prior to UVB exposure resulted in abrogation of CPDs formation, whereas large size AgNPs (60 and 100 nm) did not show any protective effect. Particle size and surface area are important characteristics from a biological point of view because the number of reactive groups increases with decreasing size and increasing surface area. Various studies have shown size-dependent modulation in biological effects of AgNPs. Silver nanoparticles in the sub-50 nm range exhibit increased anti-microbial efficacy [30]. Previously, silver nanoparticles having a particle size less than 50 nm have been shown to possess radical scavenging activity *in vitro* and anti‐ inflammatory activity against acute and chronic paw models of edema in mice [31]. Moreover AgNPs (size ≤ 50 nm) protected mice from gamma radiation induced body weight losses and mortality revealing its radioprotection capacity [31]. In another study the AgNPs (size ≤ 50 nm) and its complex with glycyrrhizic acid have been demonstrated to protect cellular DNA against ionizing radiation induced damages in Swiss albino mice [32]. Notably, we observed these protective effects of AgNPs against UVB-induced DNA damage at the concentration of 1 μg/mL, which did not exert any toxicity in HaCaT cells. Moreover, AgNPs in the size range tested (10–100 nm) did not show any significant differences in the viability of HaCaT cells at various concentration tested (1–10 μg/mL). Our findings on cytotoxicity of AgNPs are in consistent with the previously published studies [23, 33]. These findings thus not only provided a biological basis for chemopreventive efficacy of AgNPs, but also highlighted their safety for potential future human applications.

In another interesting finding, we observed that treatment of HaCaT cells with AgNPs prior to UVB-exposure lead to significant reduction of apoptosis. The process of apoptosis is controlled by a balance between the expression of pro-apoptotic and anti-apoptotic proteins [34, 35]. The results of this investigation show that when HaCaT cells are exposed to UVB radiation, the expression of pro-apoptotic protein (BaX) is increased while the levels of anti-apoptotic proteins (Bcl-xL, Bcl-2) decrease. Moreover, UVB exposure lead to an increased Bax/Bcl-2 and BaX/Bcl-xL ratio that is in accordance with the similar findings made in the earlier studies [23, 36]. Pre-treatment of cells with smaller size AgNPs (10 nm) neutralizes the UVB-induced apoptosis-related protein expression, whereas no such effect was observed with the bigger size (100 nm) AgNPs. This is clearly suggestive of size-dependent protective effects of AgNPs on UVB-induced apoptosis-associated protein expression, which could explain the observed reduction in apoptosis.

The results of these investigations show decisively that AgNPs have a size-dependent impact on protection of UVB-induced DNA damage and apoptosis in HaCaT cells. The size of nanoparticles has a strong impact on their interactions with living cells, influencing their cellular uptake and internalization mechanisms [16, 37]. Small size nanoparticles have a higher surface area to volume ratio, thus higher chances of interactions with cells and higher possibility to be internalized as compared to large ones [16, 37, 38]. Recently, the gene ontology (GO) analysis revealed that proteins involved in cell death and, mitochondrial activity were more affected by 20 nm AgNPs than by 100 nm AgNPs [39]. Moreover, treatment with smaller AgNPs (20 and 34 nm) modulated the expression of more genes than bigger particles (61 and 113 nm) in intestinal epithelium model [39]. In our previous study, we demonstrated enhanced internalization of AgNPs in UVB-irradiated HaCaT cells, and suggested that AgNPs may interact with DNA and various transcription factor proteins to alter their function [23]. Thus, in the light of observed biological effects, it is speculated that the increased protective effect of smaller size AgNPs (10–40 nm) might be attributed to their interactions with cells, influencing uptake efficiency, internalization pathway selection, intracellular localization and interactions with various genes and proteins. Despite enormous efforts in this area, it still remains a challenge to correlate a particular cellular response with size.

## Conclusion

In summary, this study suggests that silver nanoparticle’s size plays a significant role in protecting human skin keratinocytes from UVB-induced DNA damage and apoptosis. The protective effect is most pronounced in the size range of 10 to 40 nm. Interestingly, this effect starts decreasing when the silver nanoparticle size is greater than 40 nm and completely vanishes for silver nanoparticles of size 100 nm. Although future studies are necessary to test the efficacy of silver nanoparticles in the size range 10–40 nm in pre-clinical models, the data in this research provides strong indication that silver nanoparticles can be effective chemopreventive agents against UVB-induced skin carcinogenesis. We are confident that these efforts will result in a better understanding of the impact of size of nanomaterials on their interaction with biological systems and will be helpful in designing more advanced and efficient nano-particulate systems. Future work will focus on conducting studies to delineate the mechanism responsible for chemoprevention by specific size range of silver nanoparticles.

## Abbreviations

UV: Ultraviolet
AgNPs: Silver nanoparticles
CPDs: Cyclobutane pyrimidine dimers
ROS: Reactive oxygen species
